# Supplementary material for: Isolation and Characterization of Contemporary Bovine Coronavirus Strains
Source: Viruses. 2024 Jun 16;16(6):965. doi: 10.3390/v16060965 (PMC11209117; doi:10.3390/v16060965)
Supplement: Supplementary file 1 [file viruses-16-00965-s001.zip › viruses-3006928-supplementary.pdf]

Table S1. Primers designed for BCoV genomic sequencing. Nucleotide position is based on BCoV BC8 strain (GenBank No. OR502440).

Name	Forward Primer Sequence (5'-3')	Name	Reverse Primer Sequence (5'-3')	Source
BC-47-F	GTTAGATCTTTTCATAATCTAAAC	BC-4965-R	CATTTCCTAGACTCTTACCAAAACTT	[12]
BC-232-F	GGTCTCGAACTACACTGGGC	BC-1617-R	CGTGCTGGACTCCAATACACA	This paper
BC-1444-F	GCTTGCACTACTTGTGGTCAT	BC-2627-R	TCTGCAACTTTAGGTGGTTCAG	This paper
BC-1535-F	AGAGTGCAGCAGGTTATGGT	BC-2209-R	CCATGCAAATGCAAGAGCAGT	This paper
BC-2447-F	TTGAGCGTGGCTTGTTACATTCA	BC-7177-R	TGCCGTATACAGGGCGTAAC	This paper
BC-3180-F	AAGTGCTGGAGAGCCTTGTG	BC-3638-R	CCAGTATGCAACACACTGCC	This paper
BC-4699-F	GTTCAGAGCAATGTTGATGTTGTA	BC-9199-R	AAGAATGAAGTTGAAACAGAAGCAGT	[12]
BC-6086-F	TGAGTGGCCAACAGCTACAG	BC-7177-R	TGCCGTATACAGGGCGTAAC	This paper
BC-6891-F	CGGTTTCTTGCCGACTTTTGT	BC-8148-R	TCAATTCAAGGCCTGCCGAT	This paper
BC-8008-F	GCAACTGCGCATTCTTCTA	BC-12167-R	AGCCAAATCTGCCATACGCT	This paper
BC-9529-F	AGTGTGATAATGCATTTACAATGGCT	BC-15064-R	AAAATTTAGTGGTGCCTATAACAA	[12]
BC-12054-F	TGAGGCGTGTTCTAGTGGTTC	BC-16111-R	AGAAGAGCAGACCACGCAAG	This paper
BC-15088-F	ATGGCGGCTGGGATGATATG	BC-19122-R	ACCTCCATTACAGCCAGGAAG	This paper
BC-15886-F	GTCTTGCAATAGATGCTTATCCAC	BC-21890-R	TTGCACCATAGCCCAACTCAC	This paper
BC-16570-F	TTGTTAGCGAGCGCGAATTG	BC-17383-R	GTCTGGCCCTAAGCAACACA	This paper
BC-16806-F	AGTGCTCCTACGCTTGTGC	BC-22058-R	TTCCCATCCAGTGGCTAACT	This paper
BC-19259-F	ACGCCTTGCGTGTATATGGA	BC-19962-R	CTTGTCCACCACTACGCCAT	This paper
BC-19731-F	GATTATGCTAGAGAAAGTATATTTTG	BC-25050-R	TGTTGTGCATAAACAACATCATGA	[12]
BC-21977-F	CACTATCAACCCCTCATCAC	BC-26551-R	GAGGTGGCAGCCAAAGTGTA	This paper
BC-21981-F	ATCAATCCCTCATCACCGGC	BC-23429-R	TAGAGAGGCCAGACACTGCT	This paper
BC-24606-F	CCGACGTATACCTAATCTTCCCGATTG	BC-30936-R	CTTCCCCTTGGGCACTTGTCGGCA	[12]
BC-26455-F	ACTGGAGGTGCCGAAATT	BC-30196-R	CTTCTGGCGGGGCTTATTCA	This paper
BC-30103-F	TTGCTAGTCTTGTTCTG	BC-PolyA-R	TTTTTTTTTTTTTTTTGTGATT	This paper

Table S2. Analysis of the S protein amino acid variations between four pairs of respiratory and enteric strains (each pair of samples was from the same animal; E: strains from enteric samples, R: strain from respiratory samples).

Position in S protein	Consensus Amino Acid	OR502440/ BC8/E (Animal 1)	OR502442/ BC18/R (Animal 1)	EF424615/ E-AH65/E (Animal 2)	EF424617/ R-AH65/R (Animal 2)	AF391541/ ENT/E (Animal 3)	AF391542/ LUN/R (Animal 3)	EF424619/ AH187/E (Animal 4)	EF424620/ AH187/R (Animal 4)
24	V	*	*	L	*	*	*	*	L
35	S	F	F	*	*	*	*	*	*
45	N	*	*	*	*	*	K	*	*
113	I	*	*	*	V	*	*	*	*
174	P	S	S	*	*	*	*	*	*
179	Q	R	R	*	*	*	R	*	*
370	D	*	*	*	*	Y	*	*	*
483	P	*	*	*	*	S	*	*	*
492	D	G	G	*	*	*	*	*	*
499	N	*	*	S	S	*	*	S	*
501	S	*	*	*	*	P	P	*	P
509	T	*	*	N	N	*	*	N	*
510	T	*	*	*	*	S	S	*	S
525	H	Y	Y	*	*	*	*	*	*
531	D	*	*	*	N	*	*	*	*
546	P	S	S	*	*	*	*	*	*
554	Y	H	H	*	*	*	*	*	*
571	H	*	*	Y	*	*	*	Y	Y
578	S	*	*	*	*	T	T	*	*
617	T	I	*	*	*	*	*	*	*
743	S	*	*	*	*	*	*	*	I
754	S	*	*	*	*	*	*	*	N
960	P	*	L	*	*	*	*	*	*
1052	A	*	*	*	*	*	T	*	*
1180	D	*	*	*	G	*	G	*	G
1192	N	Y	Y	*	*	*	*	*	*
1196	T	*	*	*	*	*	*	*	S
1232	M	K	K	*	*	*	*	*	*
1242	D	*	*	*	*	Y	*	*	*
